# The fast exodrift after the first surgical treatment of exotropia and its correlation with surgical outcome of second surgery

**DOI:** 10.1186/s12886-018-0722-5

**Published:** 2018-03-02

**Authors:** Won Jae Kim, Myung Mi Kim

**Affiliations:** Department of Ophthalmology, Yeungnam University College of Medicine, 170, Hyeonchung-ro, Nam-gu, Daegu, 42415 South Korea

**Keywords:** Recurrent exotropia, Exodrift, Surgical outcome

## Abstract

**Background:**

To compare the rate of exodrift after a second surgery for recurrent exotropia, in patients grouped to fast versus slow exodrift after their first surgery. To determine whether there is a correlation with surgical outcome, and to evaluate the factors associated with fast exodrift.

**Methods:**

Patients with recurrent intermittent exotropia, who underwent contralateral lateral rectus recession and medial rectus resection as the second surgery and were followed up for 24 months postoperatively between January 1991 and January 2013, were reviewed retrospectively. The patients were divided into two groups according to the rate of exodrift after the first surgery: Group F, patients exhibiting fast exodrift after the first surgery (> 10 prism diopters [PD] before postoperative month 6); and Group S, patients exhibiting slow exodrift after the first surgery (≤10 PD before postoperative month 6). The difference in the clinical course over the 24 months after the second surgery between the two groups and factors associated with fast exodrift were analyzed.

**Results:**

In total, 106 patients with recurrent exotropia were enrolled in this study. Of these, 68 (64.2%) and 38 (35.8%) patients were included in group F and S, respectively. Group F showed more exodrift compared with groups S over the 24-month postoperative period; however, there was no significant difference in the clinical course between the two groups during that time (*p* = 0.54, repeated-measure ANOVA). In logistic analysis, immediate postoperative deviation after the first surgery was associated with fast exodrift (*p* <  0.001).

**Conclusion:**

Although patients with recurrent exotropia had shown fast exodrift after the first surgery, no significant difference in the surgical outcome was observed after the second surgery according to the rate of exodrift after the first surgery.

## Background

Recurrent or persistent exodeviation may occur in patients with intermittent exotropia following surgical treatment [1–4]. Patients with intermittent exotropia who underwent surgical treatment generally experience postoperative exodrift over time [5]. When a patient who has undergone surgical treatment shows noticeable exodeviation in a short period of time, a second surgery would be considered to restore the ocular alignment. When planning the second surgery for patients with recurrent exotropia, one question frequently arises: do patients who experience a fast rate of exodrift after the first surgery also show fast exodrift after the second surgery? To the best of the authors’ knowledge, there has been no study comparing the longitudinal clinical course of patients with recurrent exotropia after the second surgery according to the rate of exodrift after the first surgery. This study compared the surgical outcomes of patients with recurrent exotropia after the second surgery according to rate of exodrift after the first surgery to clarify whether patients with recurrent exotropia, who had experienced fast exodrift after the first surgery, will experience the same rate of exodrift after the second surgery. In addition, the factors associated with a fast rate of exodrift after the first surgery were also evaluated.

## Methods

This study was approved by the Institutional Review Board of Yeungnam University Hospital (IRB file number: 2015–11–044-007). Informed consent was waived by the board. A retrospective chart review was performed on patients with recurrent intermittent exotropia who had undergone contralateral lateral rectus recession and medial rectus resection (R&R) as a second surgery between January 1991 and January 2013. Patients who had undergone unilateral R&R as the first surgery were included in the study. Patients with at least a 24 months’ follow-up after the second surgery were included. The basic type, which was defined when the difference between the distant and near angle was within 10 prism diopters (PD), was included in this study. Patients included in this study were divided into two subgroups according to the rate of exodrift after the first surgery: group F comprised patients with recurrent exotropia who exhibited fast exodrift after the first surgery (> 10 PD before postoperative month 6); and group S comprised patients with recurrent exotropia who exhibited slow exodrift after the first surgery (≤10 PD postoperative month 6).

The clinical characteristics and surgical outcomes after the second surgery were compared between group F and S. Patients with any other types of strabismus, such as oblique muscle dysfunction, dissociated vertical deviation, A-V pattern, and nystagmus, were excluded. Patients with previous intraocular surgery, any neurological impairments, such as cerebral palsy, or severe unilateral amblyopia were also excluded.

### Patient evaluation and surgical plan

The patients underwent a complete ophthalmologic examination preoperatively and postoperatively, which included visual acuity testing, ocular alignment status, slit-lamp biomicroscopy, refraction, fundus examination, and stereoacuity test. The onset of exotropia was assessed using the parental or patients’ report. The patients were asked to bring old photographs if they were unable to remember the onset of exotropia. The best-corrected visual acuity was measured where possible. Amblyopia was defined as an interocular difference in visual acuity of two or more lines. If amblyopia was detected, occlusion therapy was performed to treat the amblyopia as soon as possible before surgery. The angle of deviation was measured by alternate prism cover testing at 6 m (distance fixation) and 33 cm (near fixation) in cooperative children both pre- and postoperatively. An additional near measurement was made after 1 h of monocular occlusion of the non-dominant eye or by habitually deviating the eye to measure the largest angle of deviation. The post-occlusion near measurement was obtained with an additional + 3.00 diopters (D) sphere over each eye before allowing the patient to regain their binocular fusional ability. The stereoacuity was measured using the Lang I test (LANG-STEREOTEST AG, Küsnacht, Switzerland) and Stereo Fly Stereotest (Stereo Optical Co., Chicago, IL, USA) when the patient could cooperate and complete the test. All surgeries in this study were performed under general anesthesia. The R&R procedure was undertaken using the surgical dose at the authors’ clinic (Table 1). The angle of deviation measured during the first follow-up visit within one week of surgery was defined as the immediate postoperative deviation. The patients were followed-up at postoperative month 1, 3, 6, and 12, and every 6 months thereafter. The postoperative angle of deviation was measured at each visit. To improve the statistical accuracy, patients who did not complete regular follow-ups during 24 postoperative months were excluded from study.

**Table 1:** Surgical dose of LR recession and MR resection

Prism diopters	Recession amounts of LR	Resection amounts of MR
25	4	3
30	4	4
35	5	4
40	5	5
45	7	5
50	8	5

*LR* Lateral rectus muscle, *MR* medial rectus muscle

### Statistical analysis

The continuous data are presented as the mean ± standard deviation, and the categorical data are presented as counts and percentages. Differences in the clinical course between the two groups over the 24-month postoperative period after the second surgery were analyzed using repeated measures ANOVA (rmANOVA). A univariate logistic regression test was conducted to examine the factors associated with fast exodrift after the first surgery. A *p*-value < 0.05 was considered as statistically significant.

## Results

### Demographic and clinical characteristics of the patients

A total of 231 patients with recurrent exotropia underwent contralateral R&R as the second surgery during the study period. Among these patients, 106 patients met the inclusion criteria. Of these, 68 patients (64.2%, 68/106) with recurrent exotropia exhibited fast exodrift after the first surgery and were included in group F. The remaining 38 patients (35.8%, 38/106) with recurrent exotropia exhibited slow exodrift after the first surgery and were included in group S. Therefore, approximately two-thirds of patients who underwent second surgery exhibited fast exodrift after the first surgery. The demographic and clinical characteristics of these patients are in Table 2. No significant differences in gender distribution, age at onset of exotropia, age at the first and second surgery, preoperative deviation at the first and second surgery, spherical equivalent refractive errors at the first and second surgery, and the result of the stereotest were observed between the two groups. The immediate postoperative deviation after the first surgery showed a significant difference between the two groups (Table 2, *p* <  0.001, unpaired t-test). The mean deviation at postoperative month 6 were 15.5 PD and 6.3 PD in group F and S, respectively. In group F, the mean deviation at postoperative month 6 was nearly one-half of mean preoperative deviation. The mean interval between the first and second surgery was 63.0 months in group F, which was shorter than that in group S (Table 2, *p* = 0.009). This may be because the fast rate of exodrift after the first surgery resulted in the earlier consideration of a second surgery.

**Table 2:** Demographic and clinical characteristics of the group F and S

	Group F (*n* = 68)	Group S (*n* = 38)	*p*-value
Gender (male: female)	30: 38	16: 22	0.503
Age at onset of exotropia, mo	21.3 ± 16.8 (65/68)	23.0 ± 13.5 (34/38)	0.617
Operated eye at first surgery (right: left)	32:36	18:20	0.568
First surgery			
Age at first surgery, yr	5.2 ± 1.6 (3–12)	5.3 ± 4.1 (3–29)	0.781
Preoperative distance deviation, PD	33.1 ± 5.1 (25–50)	32.7 ± 5.2 (25–50)	0.736
Preoperative near deviation, PD	33.4 ± 4.8 (25–50)	33.5 ± 5.6 (25–50)	0.926
SE refractive errors at first surgery, D			
Right eye	− 0.13 ± 0.95	−0.32 ± 1.03	0.349
Left eye	−0.17 ± 1.04	−0.22 ± 1.05	0.807
Immediate postoperative deviation, PD	2.5 ± 3.3 (−4 to 10)	−0.4 ± 3.2 (−9 to 6)	< 0.001
6 months postoperatively deviation, PD	15.5 ± 3.9 (12–25)	6.3 ± 3.5 (0–10)	< 0.001
Interval from first to second surgery, mo	63.0 ± 19.0 (31–151)	80.0 ± 35.8 (33–202)	0.009
Second surgery			
Age at second surgery, yr	8.3 ± 2.1	10.2 ± 5.8	0.059
Preoperative distance deviation, PD	27.3 ± 3.2 (20–35)	27.9 ± 3.7 (23–35)	0.390
Preoperative near deviation, PD	28.0 ± 3.7 (20–35)	28.2 ± 3.7 (23–35)	0.815
SE refractive errors at second surgery, D			
Right eye	−0.97 ± 1.79	− 1.53 ± 2.12	0.150
Left eye	−0.93 ± 1.57	−1.64 ± 2.31	0.094
Result of stereotest			
Lang I test, passed, (%)	56/66, (84.8)	32/36, (88.89)	0.256
Stereo Fly Stereotest ≤800 arcsec	20/25	11/14	0.611

Group F = patients with recurrent exotropia who exhibited fast exodrift (> 10 prism diopters [PD] before postoperative month 6) after the first surgery, Group S = patients with recurrent exotropia who exhibited slow exodrift (≤10 PD before postoperative month 6) after the first surgery, *PD* prism diopters, *D* dioptersm, *SE* spherical equivalent; arcsec; arcsecond

### Surgical outcome after second surgery according to the rate of exodrift after first surgery

**Fig. 1:**
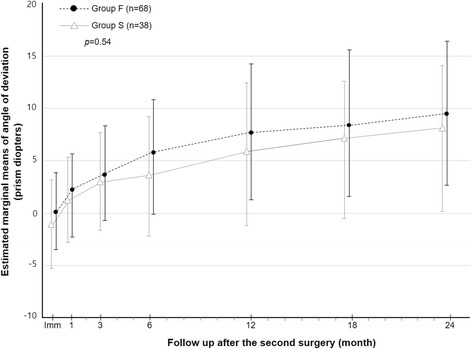
Comparison of the angle of deviation after the second surgery between group F and group S over 24 months postoperatively. The repeated measures ANOVA analysis revealed no significant difference in the group-by-time interaction in the postoperative angle of deviation between the two groups (*p* = 0.540); Group F = patients with recurrent exotropia who exhibited fast exodrift (> 10 prism diopters [PD] before postoperative month 6) after the first surgery; Group S = patients with recurrent exotropia who exhibited slow exodrift (≤10 PD before postoperative month 6) after the first surgery; Imm, Immediate postoperative

The angle of deviation in the two groups over the 24 months after the second surgery are shown in Table 3. Group F showed more mean deviation compared with group S at all postoperative follows-ups over 24-month period. However, rmANOVA analysis revealed no significant difference in the group-by-time interaction in the postoperative angle of deviation between the two groups (Fig. 1, *p* = 0.54, rmANOVA). The clinical factors associated with fast exodrift after the first surgery were evaluated between two groups. The age at onset of exotropia, gender, age at the first surgery, preoperative deviation, spherical equivalent refractive errors, immediate postoperative deviation, and the results of the stereotest were analyzed by univariate logistic analysis. Immediate postoperative deviation was the only factor to show an association with the fast exodrift after the first surgery (odds ratio: 1.352, *p* <  0.001, logistic regression test).

**Table 3:** The angle of deviation after the second surgery at each follow-up visits in group F and group S

	Group F (*n* = 68)	Group S (*n* = 38)
Immediate, PD	0.0 ± 3.7	−1.1 ± 4.1
1 mo	2.0 ± 4.3	1.1 ± 4.1
3 mo	3.5 ± 4.5	2.9 ± 4.7
6 mo	5.7 ± 5.8	3.5 ± 5.6
12 mo	7.5 ± 6.5	5.7 ± 6.8
18 mo	8.3 ± 7.1	6.0 ± 6.7
24 mo	9.4 ± 6.9	7.0 ± 6.9

Group F = patients with recurrent exotropia who exhibited fast exodrift (> 10 prism diopters [PD] before postoperative month 6) after the first surgery, Group S = patients with recurrent exotropia who exhibited slow exodrift (≤10 PD before postoperative month 6) after the first surgery, *PD* prism diopters

## Discussion

The result of this study showed that patients exhibiting fast exodrift after the first surgery were not more likely to exhibit fast exodrift after the second surgery. Patients with exotropia usually experience postoperative exodrift over time. The rate of postoperative exodrift varies among patients, with some exhibiting faster exodrift than others [5–7]. Park and Kim [7] reported drift rates over 12 months of postoperative follow-up; they indicated that drift rate was fastest at postoperative weeks 1–3, and showed the strongest correlation with overall drift rate. Additionally, previous studies [8, 9] showed that more than one-half of the total amount of postoperative exodrift occurred during the first postoperative year. Therefore, fast postoperative exodrift would occur relatively early period after the surgical treatment of exotropia. Those results are consistent with the results of the present study, which showed that mean deviation observed at postoperative month 6 in group F was approximately one-half of the mean preoperative deviation.

If fast exodrift occurs after surgery, a second surgery to restore ocular alignment can be considered. Whether patients with recurrent exotropia who had experienced fast exodrift will exhibit fast exodrift after the second surgery is of concern; however, a search of the literature did not reveal any study that evaluated the surgical outcomes of recurrent exotropia after the second surgery according to the rate of exodrift after the first surgery. This study investigated the surgical outcome after the second surgery in patients with recurrent exotropia according to the rate of exodrift after the first surgery. The patients with recurrent exotropia were divided into two groups according to rate of exodrift after the first surgery. The fast exodrift after the first surgery was defined as more than 10 PD at 6 months after the first surgery. This value was selected because most studies evaluating surgical outcome of exotropia use 10 PD as the reference in their definition of successful alignment [2–4, 10]. Because patients with recurrent exotropia may exhibit exodrift over time after the second surgery [2, 3], this study included patients who were followed up for at least 24 months postoperatively to investigate long-term surgical outcomes after the second surgery and compare these outcomes between patients with fast and slow exodrift after the first surgery.

In this study, there was no significant difference in the surgical outcome after the second surgery according to the rate of exodrift after the first surgery. Even when fast exodrift occurred after the first surgery, similar surgical outcomes were not necessarily seen after the second surgery. The results of the present study indicate that a second surgery may be considered for recurrent exotropia even though fast exodrift occurred after the first surgery. We suspect that both motor and sensory improvements after surgery were the reason that no significant differences in surgical outcomes were seen after the second surgery. From a motor perspective, the mechanical force from the resected medial rectus muscle in both eyes is considered one of the reasons for result of this study [11]. Kim and Kim [12] reported that the clinical course after a second surgery for recurrent exotropia was improved compared with the clinical course of both recurrent exotropia after the first of two surgeries and exotropia after a single surgery. From sensory perspective, even though fast exodrift occurred after the first surgery, it was assumed that there would be an improvement in the fusional ability compared with that before surgical treatment. Previous studies revealed an improvement in binocularity after surgical treatment in patients with exotropia, even constant exotropia [13–17]. These improvements in both motor and sensory aspects might lead to similar exodrift outcomes after the second surgery between the two groups.

Univariate analysis of the associated factors related to fast exodrift after the first surgery revealed its association with immediate postoperative deviation. This is consistent with a previous studies, which reported that the rate of exodrift correlated with the initial postoperative overcorrection [6, 7, 10]. However, the result of this study might be interpreted differently from previous studies, because previous studies evaluated exodrift in patients with exotropia who underwent a single surgery. This present study included only patients with recurrent exotropia who underwent a second surgery. The immediate deviation after the first surgery reached a statistical difference between the two groups, but these differences are very small from a clinical perspective. In group S, the mean deviation 6 months after first surgery progressed to 6.3 PD, only one-half of that in group F, but all included patients underwent a second surgery. Therefore, immediate postoperative deviation shows an association with fast exodrift after the first surgery, but it may not guarantee good surgical outcome over the long term.

The present retrospective study has some limitations. The mean interval between the first and second surgeries differed for each group, likely because fast exodrift after surgery can lead to earlier consideration of a second surgery. This study only included patients who underwent an R&R procedure as the first surgery. Another common surgery for the treatment of exotropia is the bilateral lateral rectus recession (BLR) procedure, and exodrift after the BLR procedure and its effect on surgical outcome after second surgery should also be determined. In addition, all surgeries were performed at the same institution and by the single surgeon. A future prospective study based on multiple institutions with a fixed interval between the first and second surgery and including both the R&R and the BLR procedure will provide more information on the clinical course of recurrent exotropia.

## Conclusions

In conclusion, surgical outcomes in patients with recurrent exotropia who exhibited fast exodrift after the first surgery were not significantly different from the outcomes in those who exhibited slow exodrift after the first surgery. In addition, immediate postoperative deviation after the first surgery was associated with fast exodrift. These results will be helpful in explaining the expected prognosis after a second surgery to patients with recurrent exotropia who had previously experienced fast exodrift.

## Abbreviations

BLR: Bilateral lateral rectus recession
R&R: Lateral rectus recession and medial rectus resection
